# Bronchiolitis Obliterans in Unicentric Castleman Disease Without Paraneoplastic Pemphigus Requiring Bilateral Lung Transplantation

**DOI:** 10.7759/cureus.92458

**Published:** 2025-09-16

**Authors:** Toyoshi Yanagihara, Hidenao Kayawake, Kentaro Tsuji, Akira Matsumoto, Takato Ikeda, Yuki Shundo, Naoki Hamada, Hiroshi Date, Masaki Fujita

**Affiliations:** 1 Department of Respiratory Medicine, Fukuoka University Hospital, Fukuoka, JPN; 2 Department of Thoracic Surgery, Kyoto University Hospital, Kyoto, JPN; 3 Department of Diagnostic Pathology, Kyoto University, Kyoto, JPN

**Keywords:** bronchiolitis obliterans, castleman disease, lung transplantation, paraneoplastic pemphigus, unicentric castleman disease

## Abstract

Bronchiolitis obliterans (BO) is an uncommon but life-threatening complication of unicentric Castleman disease (UCD), typically linked to paraneoplastic pemphigus. We report a 25-year-old woman who developed progressive dyspnea and severe, irreversible airflow obstruction in the absence of clinical or serologic evidence of paraneoplastic pemphigus. Unresponsive to inhaled triple therapy and systemic corticosteroids, she progressed to end-stage respiratory failure. She underwent living-donor bilateral lung transplantation with subsequent excision of the retroperitoneal UCD lesion. Explanted lungs confirmed concentric obliteration of small airways consistent with BO. Post-transplant, she demonstrated marked functional improvement with no recurrence of BO or UCD. This case highlights that BO can occur without detectable paraneoplastic pemphigus autoantibodies and that lung transplantation remains the only curative option for the end-stage disease.

## Introduction

Castleman disease (CD) is a rare lymphoproliferative disorder divided into unicentric Castleman disease (UCD)--typically cured by excision--and multicentric Castleman disease (MCD), which requires systemic therapy [1]. Although UCD has a favorable prognosis post‐resection, it can trigger bronchiolitis obliterans (BO), a progressive fibrosing obliteration of small airways [2-5]. BO occurs in a small subset of CD patients, most commonly in UCD, and often coexists with paraneoplastic pemphigus, an autoimmune mucocutaneous syndrome driven by autoantibodies against epidermal adhesion proteins [2,5]. Strikingly, BO may persist or even arise after complete removal of the Castleman lesion, suggesting that an autoimmune cascade becomes self-sustaining once triggered [4]. Established BO in this setting shows a poor response to standard medical treatments. In end-stage cases, bilateral lung transplantation is the only curative option.

## Case presentation

**Figure 1:**
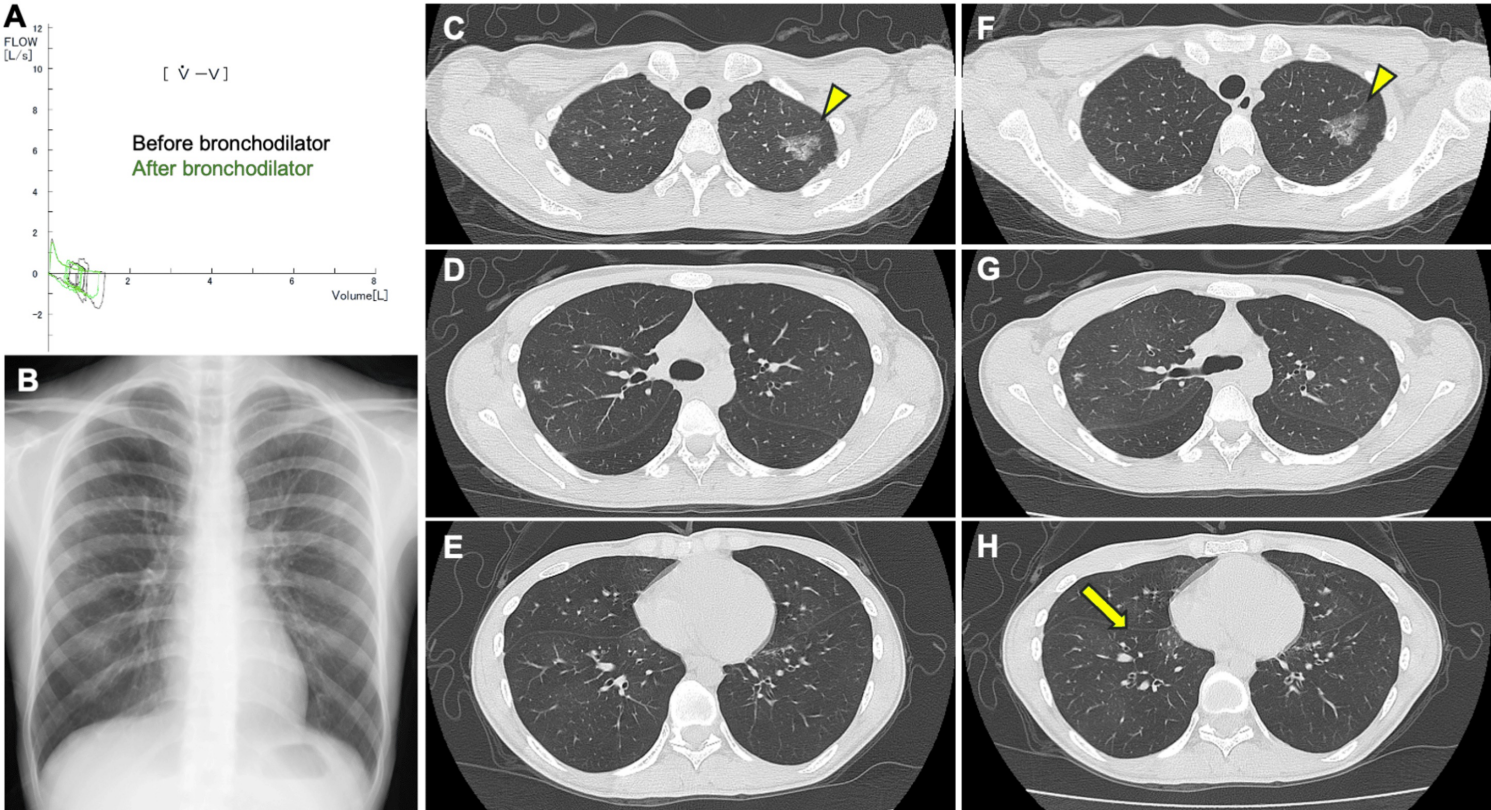
Pulmonary imaging and spirometry findings (A) Flow–volume curves before and after bronchodilator administration. (B) Chest X-ray imaging of the patient at initial presentation. (C–E) Initial CT showing focal ground-glass opacities (arrowheads) and small nodules predominantly in the bilateral upper lobes. (F–H) Expiratory CT images demonstrating areas of air trapping (an arrow) especially in the bilateral lower lobes.

**Figure 2:**
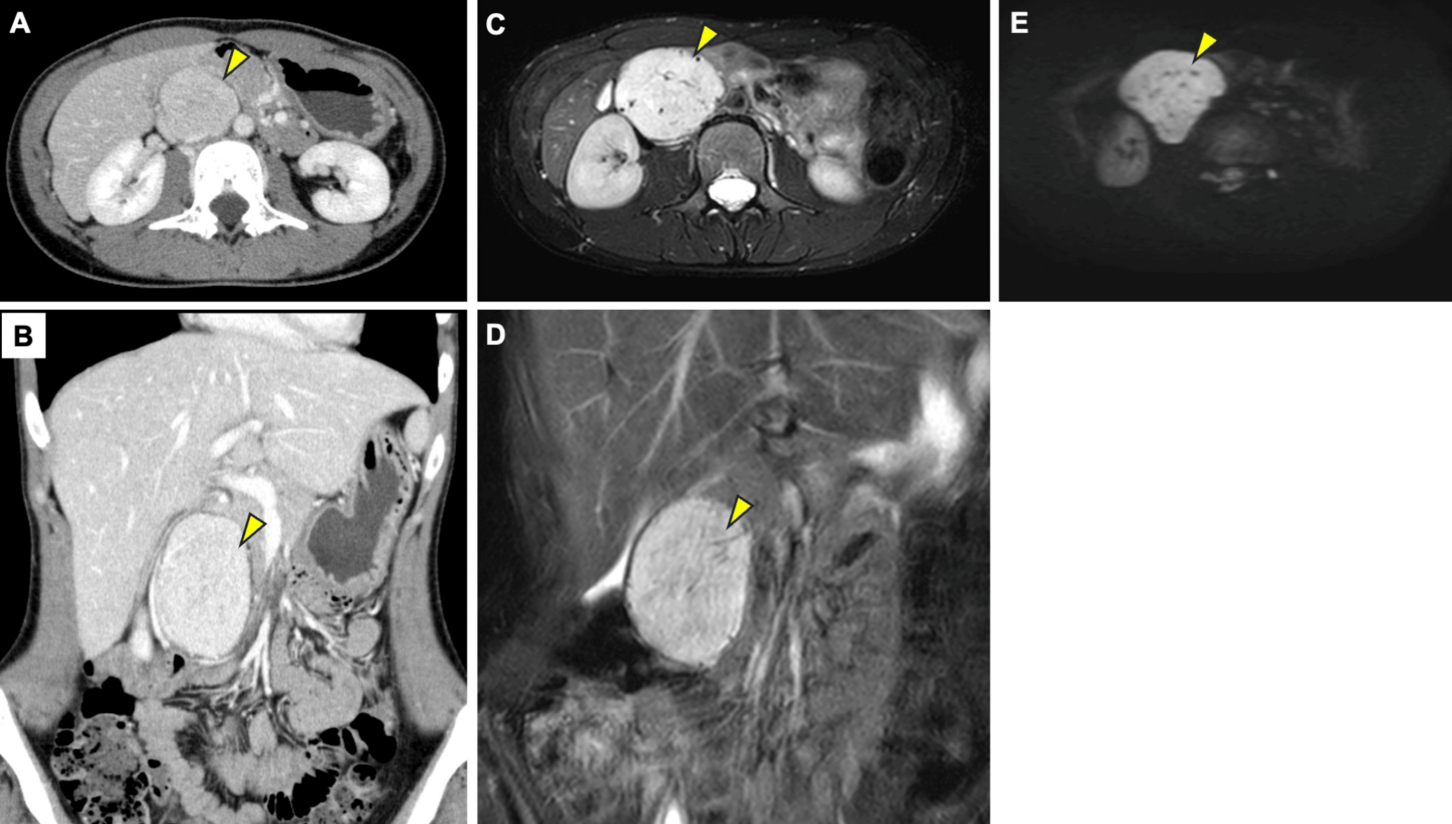
Abdominal imaging of the patient (A, B) Contrast-enhanced CT in the arterial phase demonstrates a well-defined mass (arrowheads) situated between the abdominal aorta and inferior vena cava, with intense enhancement. (C, D) T2-weighted MR images (axial and coronal) show the retroperitoneal tumor (arrowheads) as uniformly hyperintense with internal flow-voids suggestive of dilated vessels, with internal dilated vessel-like flow voids. (E) Diffusion-weighted MR image confirms high signal intensity within the lesion (an arrowhead).

A 25-year-old woman underwent bilateral lung transplantation for end-stage BO associated with UCD. Approximately four years before transplantation, at age 22, she developed a persistent dry cough and dyspnea on exertion (mMRC (Modified Medical Research Council) grade 3). Inhaled fluticasone furoate/umeclidinium bromide/vilanterol and a leukotriene-receptor antagonist provided minimal relief. Around three and a half years prior, pulmonary function tests indicated severe mixed ventilatory impairment (forced vital capacity (FVC) 1.53L (44% predicted), forced expiratory volume in one second (FEV1) 0.68L (22% predicted), FEV1% 44%, % residual volume/total lung capacity of 203%). Despite inhaled fluticasone furoate/umeclidinium bromide/vilanterol, FEV₁ did not improve on repeat spirometry (FEV1 0.6 L) (Figure 1A). Fractional exhaled nitric oxide (FeNO) was 5 ppb. Blood tests showed no eosinophilia, and immunoglobulin E (IgE) was low at 14.7 IU/mL. Initial chest X-ray identified a nodule in the upper right field (Figure 1B). Chest CT imaging showed patchy ground-glass opacities mainly in the upper lung of both sides (Figures 1C-1E). Expiratory breath CT imaging showed air-trapping (Figures 1F-1H). Contrast-enhanced CT imaging showed the abdominal tumor located between the inferior vena cava and the abdominal aorta, with a maximum enhancement of the tumor in the arterial dominant phase, and subsequent washout of contrast effect (Figures 2A-2B). Abdominal MRI revealed a retroperitoneal tumor with a maximum diameter of 77 mm, showing high signal intensity on T2-weighted imaging (T2WI), with internal dilated vessel-like flow voids (Figures 2C-2D). It was mildly hyperintense compared to the muscle on T1-weighted imaging (T1WI). It showed high signal intensity on diffusion-weighted imaging (DWI), with an apparent diffusion coefficient (ADC) value around 1,100 (Figure 2E). A planned biopsy under general anesthesia was deferred due to severe obstruction and impairment.

The severe mixed ventilatory impairment that could not be solely explained by bronchial asthma raised clinical suspicion of BO. Furthermore, the possibility of CD was considered due to the association between the retroperitoneal tumor and potential BO. Serologically, sIL-2R was 527 U/mL, and anti-nuclear antibodies were 80 titer with non-specific patterns. Rheumatoid factor and commercially measurable autoantibodies were all negative (Table 1). Serologic evaluation for paraneoplastic autoantibodies, including indirect immunofluorescence on human skin, salt-split skin, and rat bladder, immunoblotting for envoplakin, periplakin, desmogleins, and ELISA for BP180/BP230, was performed by an external medical laboratory and was entirely negative. The patient also lacked mucocutaneous lesions.

**Table 1:** Laboratory findings of the patient Comprehensive laboratory test results are summarized with corresponding reference ranges for interpretation. Abbreviations: WBC, white blood cell; RBC, red blood cell; Hb, hemoglobin; Plt, platelet; TP, total protein; Alb, albumin; CRP, C-reactive protein; AST, aspartate aminotransferase; ALT, alanine aminotransferase; BUN, blood urea nitrogen; Cr, creatinine; Na, sodium; K, potassium; Ig, immunoglobulin; sIL-2R, soluble interleukin-2 receptor; RF, rheumatoid factor; ANA, antinuclear antibody; CCP, cyclic citrullinated peptide; dsDNA, double-stranded DNA; SS-A, Sjögren’s syndrome-related antigen A; SS-B, Sjögren’s syndrome-related antigen B; Scl-70, topoisomerase I

Test	Value	Reference range
WBC (/uL)	8,500	3,300-8,600
RBC (10^4/μL)	557	435-555
Hb (g/dL)	15	13.7-16.8
Plt (10^3/uL)	288	158-348
TP(g/dL)	8.4	6.6-8.1
Alb(g/dL)	4.6	3.8-5.3
CRP (mg/dL)	0.14	0.4-1.5
AST (U/L)	24	13-30
ALT (U/L)	28	10-42
BUN (mg/dL)	11	8-20
Cr (mg/dL)	0.5	0.65-1.07
Na (mEq/L)	139	138-145
K (mEq/L)	4.7	3.6-4.8
IgG (mg/dL)	2,044	861-1747
IgA (mg/dL)	496	93-393
IgM (mg/dL)	74	50-269
IgE (IU/mL)	14.7	<232
IgG4 (mg/dL)	80	11-121
sIL-2R (U/mL)	527	121-613
ANA (dilution)	80	<40
RF (IU/mL)	7	<15
Anti-CCP(U/mL)	<0.6	<4.5
Anti-dsDNA(IU/mL)	<10	<12
Anti-SS-A (U/mL)	<1.0	<10.0
Anti-SS-B (U/mL)	<1.0	<10.0
Anti-Scl-70 (U/mL)	<0.6	<7.0

Twenty-eight months before transplant, prednisolone (30 mg/day) was trialed for respiratory failure due to suspected BO; however, no effect was observed, and prednisolone was tapered to 2.5 mg/day. Eighteen months prior, home oxygen therapy was initiated.

**Figure 3:**
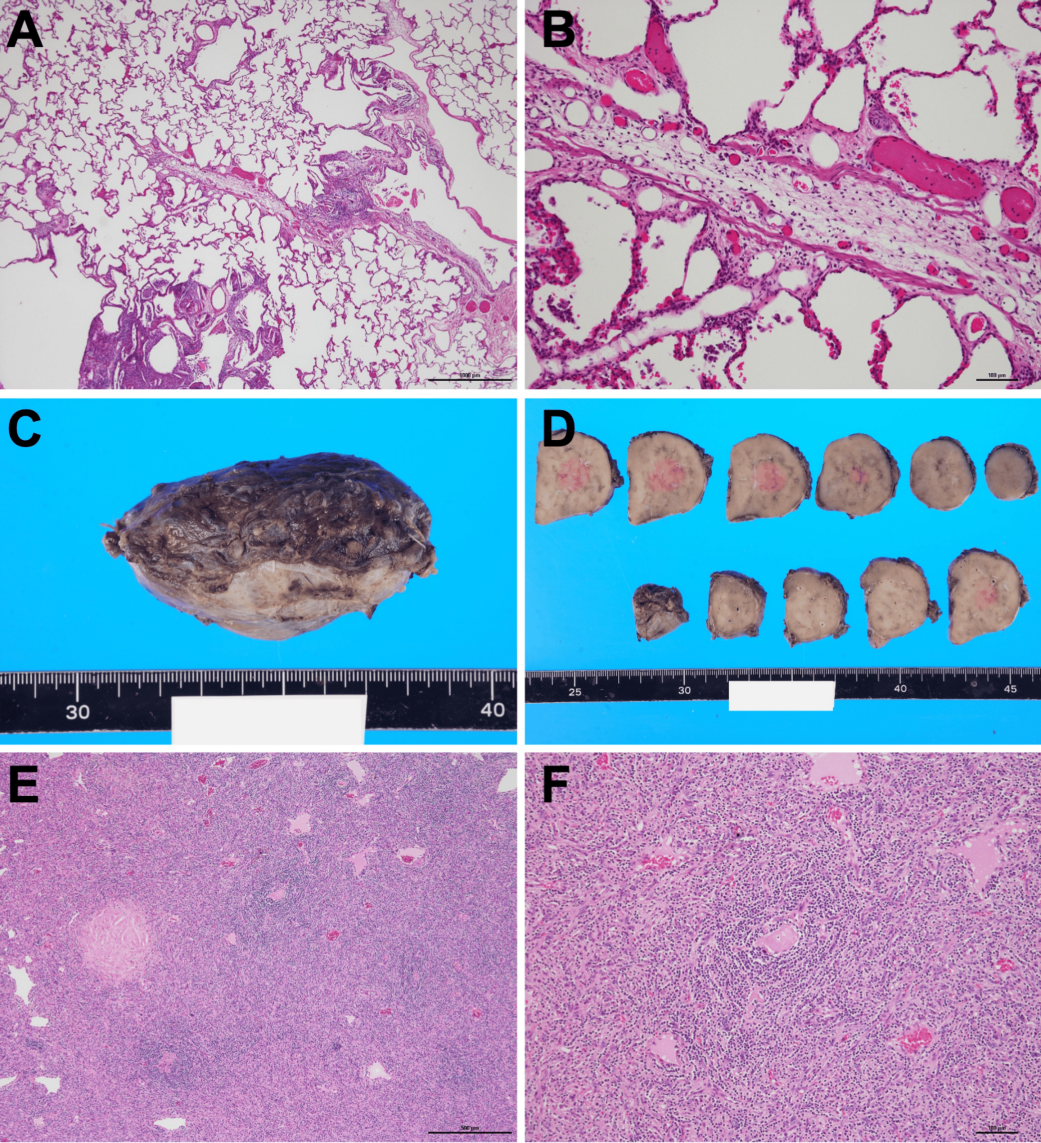
Histopathological and gross findings of the explanted lungs (A) Low-power hematoxylin and eosin stain of the explanted lung showing narrowing and obstruction of small airways accompanied by infiltration of multinucleated giant cells containing cholesterol clefts. (B) High-power view highlighting fibrous obliteration of bronchioles. This was compatible with bronchiolitis obliterans (C, D). Gross photograph of the retroperitoneal mass: a solid brown lesion with scattered microcalcifications on the cut surface. (E, F) Hematoxylin and eosin staining of the tumor showing a mixture of stromal cells and small lymphocytes, with concentric rings of lymphocytes surrounding hyalinized blood vessels.

At transplantation, living-donor bilateral lung transplantation from both parents was performed under central venoarterial extracorporeal membrane oxygenation (ECMO) without intraoperative complications. The retroperitoneal tumor was resected under general anesthesia one month after bilateral lung transplantation. Pathological examination of the explanted lungs showed features of narrowing and obstruction of the peripheral airways, extending from the membranous bronchioles to the respiratory bronchioles, accompanied by infiltration of multinucleated giant cells containing cholesterol clefts and foamy histiocytes; this was compatible with BO (Figures 3A-3B). The retroperitoneal tumor appeared as a solid, brownish lesion on the cut surface with microcalcifications (Figures 3C-3D). Histologically, it consisted mainly of stromal cells and small lymphocytes. A characteristic feature was the concentric clustering of small lymphocytes around hyalinized blood vessels (Figures 3E-3F). These findings were consistent with the hyaline vascular type of UCD. Therefore, a definitive diagnosis of UCD and associated BO was established. Postoperatively, her FEV1 is maintained at 1.33L (46.2% predicted), and she continues outpatient follow-up without recurrence.

## Discussion

Early recognition of BO remains challenging, particularly in patients without a history of prior illness or known risk factors. In such cases, clinicians should suspect BO when severe obstructive ventilatory impairment is observed that cannot be explained by asthma or other common airway diseases. Radiologically, findings that suggest small airway obstruction, such as centrilobular micronodules or the presence of air-trapping on expiratory CT, further raise the likelihood of BO. Emphasizing these clinical and radiological clues may aid in earlier detection, even in the absence of paraneoplastic pemphigus or known autoantibodies.

Most cases of BO in CD are driven by paraneoplastic pemphigus, the autoimmune syndrome that links the lymphoproliferative disorder to airway epithelial injury. Autoantibodies against desmogleins, envoplakin, and periplakin disrupt epithelial integrity in the skin and mucosa and are believed to target bronchiolar epithelium as well [6]. Typically, paraneoplastic pemphigus skin or mucosal lesions prompt serologic testing for these autoantibodies, aiding diagnosis of the paraneoplastic syndrome [6]. Importantly, however, a minority of patients, including ours, lack obvious paraneoplastic pemphigus manifestations and test negative for known paraneoplastic pemphigus autoantibodies [3,7-9]. This raises the possibility of unknown autoantibodies that specifically target bronchiolar epithelial antigens. Future research employing proteome-wide autoantibody screens may uncover previously unrecognized autoantibodies in CD-associated BO.

Beyond humoral immunity, cell-mediated mechanisms play a critical role. Histopathology of BO reveals CD8⁺ T-lymphocyte infiltrates in the bronchiolar walls, suggesting direct cytotoxic injury [2]. Furthermore, we have recently identified expansion of ST2-positive airway macrophages in a patient with BO after receiving an ﻿allogeneic peripheral blood stem cell transplant, hinting that innate immune pathways may amplify the fibrotic response [10]. Although this finding derives from non-CD settings, this suggests that similar IL-33-ST2 axis activation could contribute to tissue remodeling in CD-associated BO.

Large studies of CD-associated BO are limited. In a national Chinese cohort of 1,634 CD patients, 903 had the unicentric form, and 35 of these (3.9%) went on to develop BO [11]. To date, fewer than 20 detailed case reports have been published [3-5,8,9,12-14]. Among reported cases, the onset of BO ranged from months to years after initial CD diagnosis, and in many cases, progressed rapidly to end-stage respiratory failure despite aggressive therapy. Notably, BO may surface even after complete resection of the Castleman lesion [12], suggesting that once the autoimmune cascade is ignited, it continues independently of the tumor.

Despite medications, most patients progress to severe, life-limiting obstruction. Lung transplantation thus emerges as the only definitive therapy for end-stage BO in CD. Published series document six UCD patients undergoing bilateral lung transplant, all of whom survived surgery and experienced marked functional recovery without BO recurrence [8]. Notably, post-transplant immunosuppression appears to control any residual autoimmunity, and removal of diseased lungs may curtail the antigen presentation that drives the BO process.

## Conclusions

BO is a rare, severe complication of UCD that can occur even in the absence of paraneoplastic pemphigus or detectable autoantibodies. Early identification, based on fixed airflow obstruction and air trapping, and prompt lung transplantation offer the only path to long-term survival in end-stage disease.
